# Intravenous fluids: should we go with the flow?

**DOI:** 10.1186/cc14720

**Published:** 2015-12-18

**Authors:** Sibylle A Kozek-Langenecker

**Affiliations:** 1 Sigmund Freud Private University, Vienna, Austria; 2 Department of Anesthesia and Intensive Care, Evangelical Hospital Vienna, Hans Sachs-Gasse 10-12, 1180 Vienna, Austria

## Abstract

Sensitive monitoring should be used when prescribing intravenous fluids for volume resuscitation. The extent and duration of tissue hypoperfusion determine the severity of cellular damage, which should be kept to a minimum with timely volume substitution. Optimizing the filling status to normovolaemia may boost the resuscitation success. Macrocirculatory pressure values are not sensitive in this indication. While the Surviving Sepsis Campaign guidelines focus on these conventional pressure parameters, the guidelines from the European Society of Anaesthesiology (ESA) on perioperative bleeding management recommend individualized care by monitoring the actual volume status and correcting hypovolaemia promptly if present. The motto is: 'give what is missing'. The credo of the ESA guidelines is to use management algorithms with predefined intervention triggers. Stop signals should help in avoiding hyper-resuscitation. The high-quality evidence-based S3 guidelines on volume therapy in adults have recently been prepared by 14 German scientific societies. Statements include, for example, repeated clinical inspection including turgor of the skin and mucosa. Adjunctive laboratory parameters such as central venous oxygen saturation, lactate, base excess and haematocrit should be considered. The S3 guidelines propose the use of flow-based and/or dynamic preload parameters for guiding volume therapy. Fluid challenges and/or the leg-raising test (autotransfusion) should be performed. The statement from the Co-ordination group for Mutual Recognition and Decentralized Procedures--Human informs healthcare professionals to consider applying individualized medicine and using sensitive monitoring to assess hypovolaemia. The authorities encourage a personalized goal-directed volume resuscitation technique.

## Introduction

Organ function is dependent upon substrate flow into the tissue and outflow of waste products. Organ perfusion is generated by pressure gradients on the hydrostatic column in the vascular compartments of macrocirculation and microcirculation. Accordingly, several factors are essential for maintaining adequate tissue perfusion: pressures, filling status, vascular compartment diameter, and capillary density and permeability. This short review summarizes the rationale of the answer given in the respective presentation at the Meeting on the Future of Critical Care Medicine (FCCM) in Längenfeld, Austria, to the question of whether or not we should monitor parameters beyond macrocirculatory pressure values. Considering volume management, the answer is 'yes': we should indeed include flow-based monitoring (cardiac output) and/or dynamic preload parameters (such as stroke volume variation, pulse pressure variation) into our volume resuscitation protocols. In terms of clinicians' behaviour, the answer may sometimes be 'no'; because if we all always swim with the mainstream flow, progress and innovation would be impossible.

## Dissociation between macrocirculation and microcirculation

Blood pressure in the macrocirculation can be measured in clinical practice by non-invasive or invasive monitoring in the arterial or central venous compartments. Conventional blood pressure monitoring is well accepted among clinicians and patients, is widespread and is used worldwide. Despite the intriguing simplicity of this conventional pressure monitoring technique, it cannot assess hydrostatic and oncotic pressures at the microcirculatory level. Monitoring mean arterial pressure and central venous pressure (CVP) alone are misleading if convection and diffusion at the microcirculatory level, as well as flow, filling status and vessel capacity, are not considered. Theoretically, pressure values at the macrocirculatory and microcirculatory level do not necessarily change in parallel upon pharmacological intervention: for example, vasoconstrictors increase afterload and mean arterial blood pressure to acceptable values in the macrocirculation but may shut down downstream perfusion in the microcirculation, thus aggravating organ dysfunction. In this scenario, perfusion will get even worse in the presence of hypovolaemia and hypoviscosity. On the other hand, vasodilators decrease afterload and mean arterial pressures but may recruit capillaries and decrease diffusion distances at the microcirculatory level, thus supporting tissue perfusion and organ function. In this scenario, monitoring and optimizing the filling status to normovolaemia may boost the resuscitation success.

Techniques for microcirculatory-targeted resuscitation are on the horizon (e.g. orthogonal polarized spectral and sidestream darkfield imaging techniques, near-infrared spectroscopy, pCO_2_, contrast-enhanced ultrasonography and microdialysis). Current algorithms even incorporating microcirculatory parameters failed to improve short-term patient outcome [1]. It appears rational and highly worthwhile to proceed with clinical studies in this field.

## What do the guidelines recommend?

### Surviving Sepsis Campaign

The Surviving Sepsis Campaign [2] is increasingly criticized because of its focus on conventional management criteria: CVP 8-12 mmHg, mean arterial blood pressure >65 mmHg, urine >0.5 ml/kg/hour, central venous oxygen saturation (ScvO_2_) >70%, lactate <1.5 mmol/l and haemoglobin level >10 g/dl.

CVP remains the most widely used clinical marker of volume status, despite numerous studies showing no association between CVP and circulating blood volume [3].

### Guidelines from the European Society of Anaesthesiology

**Figure 1:**
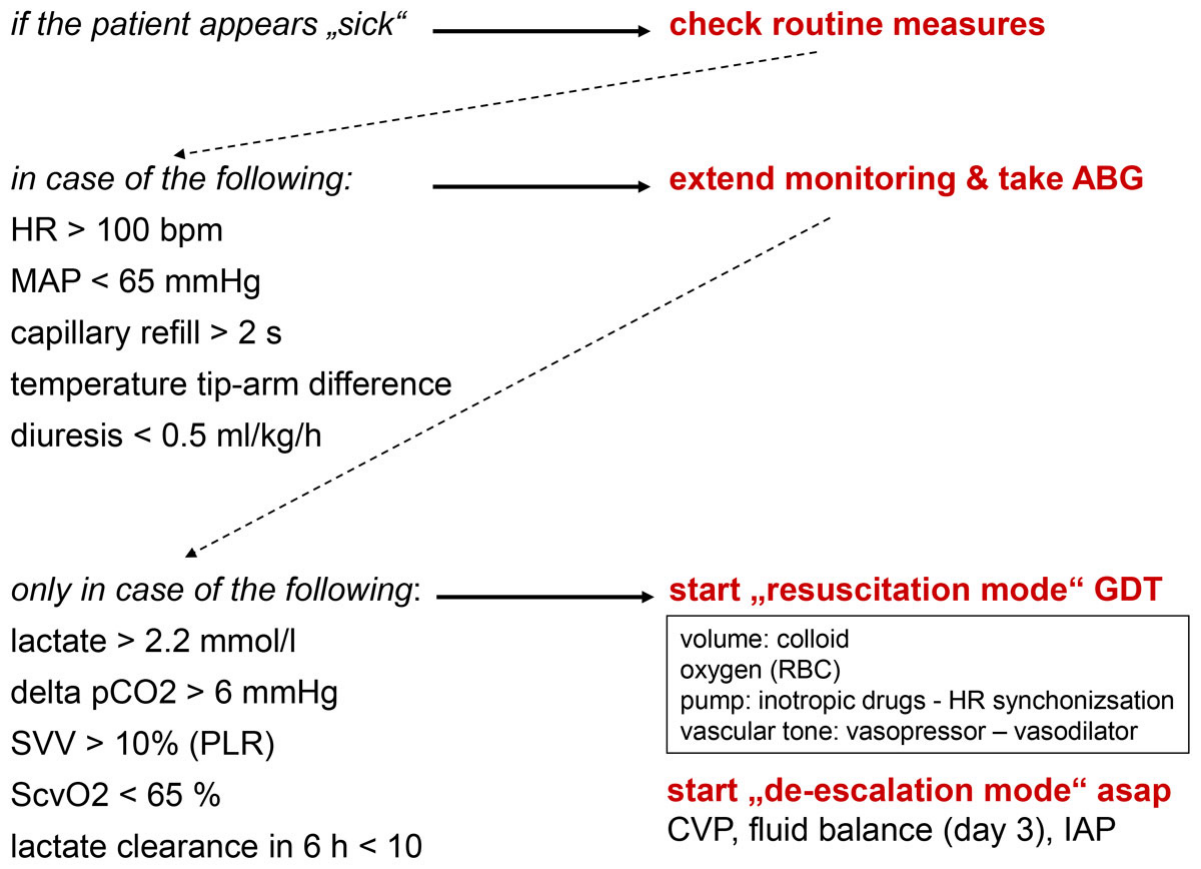
**Draft monitoring protocol for volume resuscitation at the Evangelical Hospital Vienna**. *ABG *arterial blood gas analysis, *asap *as soon as possible, *CVP *central venous pressure, *GDT *goal-directed therapy, *HR *heart rate, *IAP *intraabdominal pressure, *MAP *mean arterial blood pressure, *PLR *passive leg-raising test, *RBC *red blood cell concentrate, *ScvO2 *central venous oxygen saturation, *SVV *stroke volume variation.

The European Society of Anaesthesiology (ESA) published guidelines on the management of severe bleeding [4]. Severe blood loss leads to absolute hypovolaemia which requires volume resuscitation. Therefore, one chapter in the ESA guidelines is dedicated to the topic of infusion therapy. One key message of the ESA guidelines is to deliver individualized care by monitoring the actual status and correcting coagulopathic deficits and/or hypovolaemia promptly if present. The motto is: 'give what is missing'. The credo of the ESA guidelines is to use management algorithms with predefined intervention triggers according to another motto: 'use a protocol'. A hospital-internal draft of a management protocol is shown in Figure 1.

Some examples of respective recommendations are as follows:

• We recommend aggressive and timely stabilization of cardiac preload throughout the surgical procedure, as this appears beneficial to the patient. Grade 1B

Hypovolaemia in bleeding decreases cardiac output and tissue oxygen supply. Both the extent and duration of tissue hypoperfusion determine the severity of cellular damage, which should be kept to a minimum with timely volume substitution. The most extensively studied and successfully used method to maximize cardiac preload is the oesophageal Doppler device [5-7]. Sonographic evaluation of intravascular volume status (e.g. inferior vena cava collapsibility index) is increasingly applied at the point of care [8]. Several studies have demonstrated that dynamic parameters such as stroke volume variation or pulse pressure variation provide prediction of fluid responsiveness in mechanically ventilated patients with normal heart rhythm. Fluid challenges and the leg-raising test represent simple and valid alternatives [9]. Two recent meta-analyses concluded that a goal-directed approach to maintaining tissue perfusion reduces mortality, postoperative organ failure and surgical complications in high-risk surgical patients [10,11]. The ESA guidelines further recommend repeated monitoring of tissue perfusion, tissue oxygenation and the dynamics of blood loss during acute bleeding. Lactate clearance and base excess have been proposed as metabolic parameters for indirectly assessing tissue perfusion.

• We recommend against the use of central venous pressure and pulmonary artery occlusion pressure as the only variables to guide fluid therapy and optimize preload during severe bleeding; dynamic assessment of fluid responsiveness and non-invasive measurement of cardiac output should be considered instead. Grade 1B

This recommendation clearly describes the inferiority of sole pressure-oriented monitoring. To determine the amount of fluid required, modern monitoring techniques are necessary. The monitored variable should predict whether or not a fluid bolus will raise cardiac output.

• We recommend the avoidance of hypervolaemia with crystalloids or colloids to a level exceeding the interstitial space in steady state, and beyond an optimal cardiac preload. Grade 1B

The motto is: 'keep the bleeding patient neither too wet nor too dry'. The relationship between risks and total volume infused appears to follow a U-shaped curve: infusing too much can be as deleterious as infusing too little [12]. Fluid excess can have a negative impact not only on dilutional coagulopathy [13], but also on cardiac, pulmonary and bowel function, wound healing as well as water and sodium regulation [14]. Iatrogenic hypervolaemia predisposes patients to interstitial oedema, which appears to be associated with perioperative mortality [15]. It requires sensitive monitoring for the filling status in order to identify the 'stop signal' for further infusion therapy. An increase in CVP could be used as a stop signal.

In the ESA guidelines, monitoring technologies and their limitations are not described, monitoring-dependent intervention cutoff points are not reported and differential indications for monitoring modalities in specific clinical situations are not suggested. Clinicians have to select the most appropriate monitoring technique; for example, using the internal jugular or femoral vein collapsibility index during fluid resuscitation in patients with elevated intra-abdominal pressure or morbid obesity where sonographic acquisition of the inferior vena cava collapsibility index is not feasible [8].

### German S3 guidelines on volume therapy in adults

Recently the high-quality evidence-based S3 guidelines on volume therapy in adults have been published [16]. The German Society of Anaesthesiology and Intensive Care Medicine (DGAI) masterminded this interdisciplinary project in collaboration with 13 German scientific societies. Together, these guidelines represent the consensus among general and visceral surgeons, gynaecologists and obstetricians, urologists, internal medicine physicians and cardiologists, intensive care, neurointensive care and emergency physicians, orthopaedic and trauma surgeons, cardiothoracic and vascular surgeons, as well as nurse scientists. Eventual dissent is clearly marked in the guidelines. The S3 guidelines are currently only available in German but the translation into English is ongoing.

Statements are in line with the ESA guidelines [4]. Patient's history assessment and repeated clinical inspection of the actual volume status are recommended (e.g. by assessing turgor of the skin, mucosa) (grade of recommendation (GoR): A). Adjunctive laboratory parameters such as ScvO_2_, lactate, base excess and haematocrit should be considered (GoR: A). CVP is inadequate for detecting volume deficits in perioperative and critically ill patients (GoR: A). The German Sepsis Society disagreed with this recommendation and with a number of further recommendations which are not in line with the Surviving Sepsis Campaign [2]. The S3 guidelines propose the use of flow-based and/or dynamic preload parameters for guiding volume therapy. In high-risk patients--for example, older patients with hip-near fracture, major abdominal surgery or cardiovascular comorbidities compromising the capability for haemodynamic compensation--volume monitoring should be performed throughout major procedures (GoR: 0). Implementation of flow-based parameters into predefined management algorithms is recommended (GoR: B). Fluid challenges and/or the leg-raising test (autotransfusion) should be performed (GoR: B). If possible, volume status and responsiveness to the fluid challenge should be assessed by cardiac output monitoring or dynamic preload monitoring (GoR: B). Only initial fluid responsiveness could be roughly appreciated by changes in mean arterial pressure (GoR: 0). In intensive care patients, the S3 guidelines suggest monitoring means of intrathoracic blood volume, Doppler, B-mode and/or transthoracic echocardiography (GoR: 0). In haemodynamic instability (of suspected cardiac origin), monitoring using echocardiography is suggested (GoR: A). Echocardiography is also suggested for detecting interstitial oedema and extravasation (e.g. in the pleura, abdomen, intestine). These signs should be used as stop signals for further volume therapy.

Focus on inadequate monitoring parameters results in misleading interpretations of therapeutic interventions, and even recent randomized controlled trials used conventional static pressure-based indices as measures for haemodynamic stabilization [17,18]. The authors of the S3 guidelines therefore propose that future studies on volume management must employ adequate parameters for detecting hypovolaemia, must prove targeted correction according to a management algorithm, and must avoid overdosing.

## Societal perspective

After the statement from the European Medicines Agency (EMA) [19], hydroxyethyl starch (HES) is less prescribed Europe-wide because of medico-legal considerations. The EMA supported us to think twice before infusing any fluid intravenously and to acknowledge that fluids are drugs. Interestingly, other colloids are also increasingly being avoided although the evidence for safety problems for albumin or gelatin is scarce. In the statement from the Co-ordination Group for Mutual Recognition and Decentralized Procedures--Human (CMDh), healthcare professionals are informed to consider that 'HES solutions should only be used for the treatment of hypovolaemia due to blood loss when crystalloids alone are not considered sufficient' [19].

With this article the authorities reinforce applying individualized medicine and using, for example, preload monitoring to assess hypovolaemia. This is a step forward from conventional pressure-based management strategies. Interestingly, blood loss is defined as a prerequisite for colloidal HES infusion; replacement of extracellular water losses is clearly not listed as an indication for a colloidal infusion. The wording in this article leaves room for individual decision-making because the lack of efficacy of crystalloids does not need to be proven in the individual patient before choosing a more potent colloid, but only anticipated by the attending clinician.

'HES solutions should be used at the lowest effective dose for the shortest period of time. Treatment should be guided by continuous haemodynamic monitoring so that the infusion is stopped as soon as appropriate haemodynamic goals have been achieved [19].'

The CMDh group requires clinicians to monitor the haemodynamic status continuously, however, without defining the appropriate methodology. From a practical viewpoint, and extrapolated from the ESA and S3 guidelines [4,16], appropriate haemodynamic monitoring will most probably not be restricted to conventional pressure-oriented monitoring but will comprise volumetric preload testing. The CMDh statement reminds us about a general principle in pharmacotherapy: repeatedly reconsider the indication and avoid overdosing. The therapeutic target is to achieve normovolaemia, not infusing beyond. The CMDh statement also suggests the use of protocols with predefined triggers for infusion and as stop signals for further infusion. Thereby, the authorities encourage a personalized low-HES volume resuscitation technique. Contraindications against the use of HES, such as in sepsis and burn, are clearly listed in the EMA resolution [19].

## Goal-directed therapy

The term goal-directed therapy (GDT) in the context of volume therapy means rational, comprehensible, standardized and individualized indication and dosing of infusions. The alternative treatment option is administration of infusions at the clinicians' discretion, based on experience, intuition and gut feeling. By recommending the use of algorithms with predefined intervention triggers, S3 and ESA guidelines as well as the EMA propose GDT for volume management over non-protocolized care [4,16,19]. Since GDT has repeatedly been confirmed to reduce the length of hospital stay and postoperative complication rates, which per se determine long-term survival after major surgery [20], national authorities such as the National Institute for Clinical Excellence (NICE) in the UK facilitate top-down GDT implementation with accounting incentives.

The term GDT is not restricted to protocols incorporating flow-based parameters for guiding volume indication and dosing, but is also used for protocols with conventional macrocirculatory pressure-based parameters or microcirculatory parameters. However, using predominantly volume status-insensitive pressure parameters, clinical outcome was not improved in GDT-treated groups [21,22]. Obviously, determining the mean arterial pressure, CVP and ScvO_2 _does not increase survival. The number of trials investigating flow-based GDT is steadily increasing. First-generation algorithms aimed at maximizing stroke volume before the hit of surgery and bleeding [23]. This approach resulted in high-volume loading which often was not required during an uneventful course of surgery. Second-generation algorithms aimed for optimizing stroke volume only when found low in the presence of clinical signs of hypoperfusion, including stop signals preventing volume overload, and incorporating also protocol pathways to a rational use of vasoactive drugs [24,25]. The ideal GDT has not yet been defined--we are climbing up the learning curve. Future algorithms may personalize the GDT concept further by selecting the most appropriate monitoring technology for specific clinical scenarios; for example, in patients with active breathing efforts, with reduced left ventricular ejection fraction or with intraoperative changes in body positioning.

The ideal endpoint for clinical studies on GDT has not yet been defined either--mortality may not be adequate for the supportive therapy of volume resuscitation. In perioperative patients mortality is low, and in sepsis and critical illness mortality is affected by various pathomechanisms and interventions other than tissue perfusion and recruitment of weak microcirculatory units. It is conceivable that, despite optimized tissue perfusion, cytopathic hypoxia leads to cellular death, organ dysfunction and clinical death because failing mitochondria cannot extract delivered oxygen [26]. Intervening only on the small wheel of volume therapy in the complex sepsis syndrome cannot easily lead to success.

A direct efficacy endpoint in GDT studies could be the prevention of fluid overload. Studies have shown that a positive fluid balance is associated with increased mortality [27]. Stroke volume-directed GDT demonstrated volume efficacy of HES in neurosurgical patients: the amount of crystalloid needed in the prone position was 25% higher compared with HES [28].

National quality improvement programmes [20,29] may help us identify successful healthcare practices in real clinical life. If flow-based goal-directed volume resuscitation is adding a sustained net-clinical benefit, a signal should emerge in an outcome-oriented database. Obviously, the quality of benchmarking is dependent upon careful selection of meaningful outcome quality indicators, avoidance of missing data by automated documentation without excess workload to healthcare providers, and--most important--careful interpretation of gathered results.

## Conclusion

We should critically review a pressure-guided standard resuscitation strategy based on the Surviving Sepsis Guidelines. New evidence-based international and interdisciplinary guidelines and the EMA support the concept and advice to implement flow and preload parameters into our volume resuscitation protocols. Yes, we should go with flow-based monitoring (cardiac output) and/or dynamic preload parameters (such as stroke volume variation, pulse pressure variation) rather than only with pressure-based parameters (such as mean arterial blood pressure, CVP). Clinicians are encouraged to compile flow-based GDT and further studies are highly warranted for improving the future in critical care medicine.

## Abbreviations

CMDh, Co-ordination Group for Mutual Recognition and Decentralized Procedures--Human; CVP, Central venous pressure; DGAI, German Society of Anaesthesiology and Intensive Care Medicine; EMA, European Medicines Agency; ESA, European Society of Anaesthesiology; FCCM, of Critical Care Medicine; GDT, Goal-directed therapy; GoR, Grade of recommendation; HES, Hydroxyethyl starch; NICE, National Institute for Clinical Excellence; ScvO_2_, Central venous oxygen saturation.

## Competing interests

SAK-L received honoraria for lectures on fluid therapy from B. Braun and Fresenius Kabi. Fresenius Kabi supported the author's pooled analysis of randomized clinical trials comparing blood loss in major surgery by covering the costs for biometrical analysis performed by an independent institution. The author received no honorarium and funding for this publication.

## Notes

This article has been published as part of *Critical Care *Volume 19 Supplement 3, 2015: Future of Critical Care Medicine (FCCM) 2014. The full contents of the supplement are available online at http://www.ccforum.com/supplements/19/S3. Publication of this supplement was supported by Fresenius Kabi.
